# Incremental financial costs of strengthening large-scale child nutrition programs in Bangladesh, Ethiopia, and Vietnam: retrospective expenditure analysis

**DOI:** 10.1186/s12992-025-01118-3

**Published:** 2025-04-21

**Authors:** Tina G. Sanghvi, Rick Homan, Tuan Nguyen, Zeba Mahmud, Tamirat Walissa, Marina Nersesyan, Patricia Preware, Edward A. Frongillo, Roger Matheson

**Affiliations:** 1 FHI 360, Washington, D.C. USA; 2 FHI 360, Durham, N. Carolina USA; 3 FHI 360, FHI 360, Hanoi, Vietnam; 4 FHI 360, Dhaka, Bangladesh; 5 FHI 360, Addis Ababa, Ethiopia; 6 Department of Health Promotion, Education, and Behavior, University of South Carolina, 915 Greene St, Columbia, SC 29208-4005 USA

**Keywords:** Child nutrition, Financial costs, Complementary feeding practices, Large scale programs, Bangladesh, Ethiopia, Vietnam

## Abstract

**Background:**

Inattention to young child growth and development in a transitioning global environment can undermine the foundation of human capital and future progress. Diets that provide adequate energy and nutrients are critical for children’s physical and cognitive development from 6 to 23.9 months of age and beyond. Still, over 70% of young children do not receive foods with sufficient nutrition particularly in low-and-middle income countries. Program evaluations have documented the effectiveness of large-scale behavior change interventions to improve children’s diets, but the budgetary implications of programs are not known. This paper provides the incremental financial costs of strengthening three large-scale programs based on expenditure records from Bangladesh, Ethiopia, and Vietnam.

**Results:**

The programs reached between one and 2.5 million mothers and children annually per country at unit costs of between $0.9 to $1.6 per mother and child reached. An additional 0.7 to 1.6 million people who were influential in supporting mothers and achieving scale were also engaged. The largest cost component was counselling of mothers. Rigorous external impact evaluations showed that over 434,500 children benefited annually from consuming a minimum acceptable diet in all countries combined, at an annual cost per country of $6.3 to $34.7 per child benefited.

**Conclusions:**

Large scale programs to improve young children’s nutrition can be affordable for low- and middle-income countries. The study provides the incremental costs of selectively strengthening key program components in diverse settings with lessons for future budgeting. The costs of treating a malnourished child are several-fold higher than prevention through improved improving young children’s dietary practices. Differences across countries in program models, coverage, costs, and outcomes suggest that countries need a minimum investment of resources for strengthening high-reach service delivery and communication channels and engaging relevant behavioral levers and community support for mothers to achieve impact at scale.

**Supplementary Information:**

The online version contains supplementary material available at 10.1186/s12992-025-01118-3.

## Background

Widespread food insecurity, nutrient deficiencies, and over- and undernutrition reflect severe threats to child health, growth, and cognitive development [1]. Globalization processes affecting the environment, health systems and the social, economic, commercial, and political determinants of childhood nutrition have left millions with diminished capacities [2, 3]. Child nutrition is driven by global factors and can potentially drive global development since nutrition and dietary practices in the early stages of an individual’s life can strengthen the foundation for later health, well-being, productivity, and contributions to socioeconomic development [4]. Climate change and the COVID-19 pandemic have tested the nutritional and health resilience of children [5].

Undernutrition annually underlies nearly 3·1 million deaths of children younger than 5 years worldwide, representing about 45% of all deaths in this group; deaths can be reduced by 15% if populations can access evidence-based nutrition interventions to improve infant and young child nutrition at 90% coverage [6]. Preventive interventions have the potential to pre-empt several facets of poor nutrition during the critical first two years of life through improving children’s diets [7]. A lack of documentation, however, on what it costs in different contexts to achieve better child nutrition on a large scale has delayed progress [8]. This paper aims to provide the incremental financial costs of strengthening three child nutrition programs based on expenditure records from Bangladesh, Ethiopia, and Vietnam where interventions were implemented on a large scale and rigorously evaluated [9–11]. The intended audience includes technical specialists and generalists who are engaged in planning or implementing large-scale child nutrition programs.

How and what infants and young children are fed have serious long-term implications for growth and survival due to heightened nutritional needs for rapid physical growth and brain development; it is considered a crucial window for establishing a lifelong foundation for optimal health and neurodevelopment [12, 13]. Infants and young children need complementary foods in addition to continued breastfeeding from six to 24 months of age [7]. Supporting 90% of children to receive the recommended infant and young child feeding (IYCF) practices in low- and middle-income countries (LMICs) is estimated to save 823,000 lives annually; reduce stunting, which affects 160 million children globally; and increase years of completed schooling and their Intelligence Quotient and earnings in adulthood [14–17]. Achieving a high prevalence of recommended complementary feeding (CF) practices in a young child population is a challenge for many countries [3]. Nutrient densities of family diets often fed to children particularly in LMICs are inadequate in relation to the energy and nutrient requirements of children in the 6 to 23.9 months age group [18]. The United Nations-led Global Action Plan to Reduce Wasting highlighted the importance of improving CF as one of four pillars that are urgently needed to address wasting, which carries a high risk of child mortality and morbidity [19]. Recently, a rising trend has also been documented in the feeding of ultra-processed foods to children that contain unsuitable or harmful ingredients [20].

To improve deleterious CF practices, several interventions have been developed and assessed [21–24]. Effective behavior change interventions alone with no provision of food or cash transfers were found in a meta-analysis to improve the uptake of recommended foods by 62% (RR 1.62; 95% CI: 1.17, 2.26) [25]. These reviews were mostly based on efficacy studies or small-scale interventions with limited evidence of population-level impact from evaluations of large-scale programs [26]. Also, the costs of large-scale programs and what to budget for effective programs are still not well understood. Previous studies on the costs of child nutrition programs combined multi-sectoral and food supplementation interventions with behavior change interventions [8, 27, 28]. The literature provides few studies on the costs of comprehensive large-scale programs for improving CF practices. These limitations are due to the small scale of many of the programs studied and limited details on interventions and costing methods. The AIN-C program in Honduras combined monthly weighing and counselling sessions, and a hypothetical program based on this model was estimated to cost $ 6.43 annually per participating child [29]. A nutrition education intervention integrated into facility-based child health services in Peru estimated the incremental financial cost per child reached to be US$6.12 [30]. As expected, adding a product to the package of child nutrition interventions increased unit costs. A study comparing costs of community and facility-based distribution of micronutrient powder for children in rural Uganda estimated the community platform to cost $53.24 per child reached as compared with $65.97 per child reached for the facility platform [31]. The cost per child consuming the product was $58.08 for the community platform and $72.69 for the facility platform. Product costs were 24% of total costs. The estimated cost of scaling up the community platform pilot to the district level to cover approximately 17,890 children was $1.23 million if integrated into an NGO program or $1.62 million through a government program. The authors added 17 to 20% in opportunity costs for unpaid village health workers, and other participants.

A multisectoral nutrition program in Bangladesh that integrated behavior change communication and agricultural extension with a credit platform to support women’s income generation reported the financial costs were $598,578 over 3.5 years, and the program reached 11,110 adults and children; the incremental financial cost was $15.40 per person reached per year [32]. Combining multiple sectoral programs appears to increase costs particularly when food distribution is involved. A study of two food-assisted maternal and child health and nutrition programs targeted to pregnant women and children below 2 years found that providing households with food rations during pregnancy and up to the child’s second birthday along with behavior change and improved health services cost approximately $1,080 per participant in Guatemala and $770 per participant in Burundi [27]. The prohibitive cost of a child becoming malnourished because of inadequate CF can be observed from costing studies on treating malnourished children. In Pakistan, the out-patient care provided by Lady Health Workers cost was $291 and $382 per child treated and recovered, respectively, including beneficiary costs [33]. A study in Bangladesh compared costs of community health worker treatment of malnourished children and found they ranged across program models from $145 to $180 and $165 to $203 per child recovered and per child treated, including beneficiary costs [34].

These studies did not indicate costs of programs that could alleviate barriers to CF using social behavior change to optimize existing family resources [25, 35, 36]. There are missed opportunities in improving CF that do not require provision of costly supplements or establishment of complex new multisectoral platforms [23, 37]. This paper aims to address the critical gap regarding the cost of implementing CF programs at scale that is key for budgeting national programs. Information on costs supports decision-makers in further improving and expanding urgently needed CF programs [8]. The aim of this analysis is to estimate the incremental financial costs of developing and implementing large-scale CF programs based on actual program expenditures in Bangladesh, Ethiopia, and Vietnam. The novelty of this article lies in providing actual expenditures incurred for improving child nutrition through well-documented large-scale programs that showed positive impacts in three quite different settings by using innovative ways to integrate proven interventions in existing service delivery and communication platforms at national, health center, and community levels. Our findings will provide needed insights into the budgetary requirements of large-scale CF programs for priority-setting and targeting resources by governments and for motivating donor investments in improving human capacity development through child nutrition.

## Methods

This analysis is based on the first five years of implementation of a nutrition initiative with a focus on infant and young child feeding to save lives, prevent illnesses, and contribute to healthy growth and development in LMICs from 2009 to 2014. The information provided in this article is intended to support the planning and implementation of improved large-scale child nutrition programs, specifically for complementary feeding during 6 to 23.9 months of age. Bangladesh, Ethiopia, and Vietnam were selected for their large populations and different contexts. The costs represent expenditures on additional activities layered onto existing services to generate impacts on nutrition practices. The cost to participants is not included. Therefore, these costs are incremental expenditures incurred to enable/facilitate significant improvements in existing programs. In collaboration with national authorities and as noted in country policies and strategies, six CF practices were prioritized in all programs: timely introduction of foods at 6 months; dietary diversification through the use of four or more specified nutrient-rich foods daily; meal frequency of 2–3 times a day at 6–8 months and 3–4 times a day at 9 months or older; discouraging unhealthy foods; continued feeding and fluids during illness and increased feeding after illness; and breastfeeding through two years of age [38, 39]. In this analysis we focus on dietary diversity combined with recommended meal frequency as a single indicator for assessing adequate diets of young children [7].

For costs, we used a top-down, gross costing method based on actual expenditures incurred in each county program to estimate the incremental financial costs of the three large-scale CF programs in US dollars ($). The costs represent a payer perspective and include expenditures incurred for CF over and above existing activities and personnel already working in program areas. We extracted data on financial expenditures from records maintained by each country-specific program’s accounting database. The methodology is an accounting of financial expenditures rather than an economic analysis of costs [40]. We compared the total costs, average total costs per year, costs of program components, and cost per participant in CF programs implemented in each country. The data sources for costing were similar across the countries and involved researching expenditure records from a standardized accounting system maintained by the overall initiative. Standardization was maintained in the countries’ accounting records for categorizing expenditures on CF programs and allowed us to make cross-country comparisons of total and program component costs. Details on the programs are provided in Additional File 1.

### Calculating incremental financial costs

We extracted data on payments made to field implementing agencies for various tasks such as formative studies and assessments used for developing and planning activities; designing, printing, and disseminating tools and materials; building the capacity of frontline workers; providing supervision and monitoring; conducting community events; and public education and mass media design and dissemination. We entered information on invoices paid to implementing partners into our accounting database. All expenditures were converted by accounting managers to $ using the prevailing exchange rates. Conversion into 2022 $ was done using the Consumer Price Index ratio of the year of expenditure to the Consumer Price Index in 2022 using data obtained from the World Bank World Development Indicators available online at https://data.worldbank.org/products/wdi. We aggregated the cost components to calculate the total incremental costs of each country’s program. Program reports provided the total program durations, and the total costs incurred over the life of each country program were divided by the number of project years to calculate the average annual costs per year. We calculated costs per program component per country, across the three countries.

### Calculating the number of participants

The number of participants consists of the number of mothers and children 6-23.9 months of age who were reached by the two main categories of interventions interpersonal communication (IPC) or counseling and public education and mass media (PE/MM). Participants were calculated from program coverage results and population figures.

We use three measures of participants as the denominator for estimating coverage: (1) number of mothers and children 6 to 23.9.9 months of age residing in areas of intensified implementation (where the evaluations were conducted), other areas where the same model of IPC was adapted and implemented, and areas reached through PE/MM; (2) all individuals reached that were needed to produce an impact consisting of mothers and children 6 to 23.9 months of age based on coverage evaluation surveys and influential individuals, including family and community members, peers and health personnel; and (3) number of children 6 to 23.9 months of age in IPC areas with improved CF practices as measured through evaluations using minimally adequate diet (MAD) as the indicator (WHO 2008).

#### Number of mothers and children reached

Program records, monitoring data and impact evaluation results provided coverage percentages for each country. For exposure to public education/media, we used the percentage of mothers who recalled seeing or hearing messages on CF from any public education or media channel. For IPC, we measured the percentage of mothers who recalled being counselled or attending community groups/events where CF was discussed face-to-face with mothers. Data sources for calculating participants reached by country are shown in Additional File 2. The numbers of mothers and children under two years were those residing in program areas from program monitoring data, 2012–2014 national and regional population data, and World Bank population databases, accessed at https://data.worldbank.org/indicator/SP.POP.TOTL. Coverage was determined by cluster-randomized surveys conducted by external agencies in representative samples of program areas using standardized methods across the three countries. The coverage in scaled up or expansion areas was calculated by using control-arm coverage for public education/mass media, media monitoring reports from advertising agencies involved in implementation, process evaluation studies of service delivery, descriptions of expansion models, and monitoring and supervision data combined with implementation records.

For Bangladesh, data from household census of mothers and children collected by national health services combined with program coverage from evaluation surveys showed that 1.7 million mothers of children < 2 years were reached in 50 upazilas over 2 years [41], with a coverage of 92% for IPC, 73% for PE/MM (coverage measured in program areas), and 67% PE/MM coverage measured in control or non-IPC program areas [10]; 74% is the 20% discounted IPC coverage in scaled up areas based on comparing details of the intensive and scaled-up program implementation models [42].

For Ethiopia, data from household census of mothers and children collected by national health services combined with program coverage from evaluation surveys showed that 1.5 million mothers of children < 2 years were reached in 295 woredas over 2 years [41, 43]. About 38% of mothers were measured through cluster-randomized evaluation surveys as having received IPC, with 30% coverage of PE/MM found through evaluation surveys in 89 intensive areas [9]; a lower coverage reduced by 40% is used to calculate coverage in low-intensity expansion areas based on a process evaluation study showing lower fidelity to the program model in expansion areas [44].

For Vietnam, data from household census of mothers and children collected by national health services combined with program coverage from evaluation surveys showed that 2.3 million mothers of children < 2 years were reached nationally over 3 years [45]. The catchment area population of CHCs where the intervention was located provided the numerator; for the denominator we used 42% coverage measured through cluster-randomized evaluation surveys for IPC. About 36% PE/MM coverage was measured through cluster-randomized evaluation surveys in intense areas, with 31% PE/MM coverage in low intensity areas [11]. Additionally, 30,000 mothers and children 6-23.9 months were reached through community support groups for IPC [46] with coverage of 50% according to program records.

#### Number of influential persons reached

Special efforts were made in IPC areas to engage community thought leaders such as local ‘doctors’, priests, imams, fathers, elder women, food sellers, and members of women’s networks. We included five potential influential groups, and information used for specifying and calculating those engaged in the respective programs is shown below. In all country programs, the number of health personnel trained in CF and equipped with tools was included; family members in areas reached with IPC and/or PE/MM were included. Data sources for calculating influentials reached by country are shown in Additional File 2. In all countries, formative studies conducted for program design and baseline surveys identified categories of influential persons who needed to be reached. Monitoring data and information from program implementation and supervision reports provided the basis for calculating influentials reached through program interventions such as training, community forums, home visits, and mass media. Endline evaluation surveys measured improvements among some groups, e.g., knowledge and activities of health workers, and community and family members.

#### Bangladesh

In Bangladesh, 75,000 health personnel were trained according to training lists; they were drawn from government health services, private clinics and NGO agencies, validated through observation visits, spot checks, surveys and receipts of training per diems paid, and training materials distributed. Evaluation surveys showed that in addition to three cadres of BRAC workers who delivered IYCF services, there were trained allopathic doctors, trained traditional birth assistants, government health workers, and traditional village doctors in each community who influenced practices and were reached by the program through orientation and training, community forums, and mass media.

Family members were calculated based on survey results on household characteristics and mothers’ responses to from whom they seek guidance; we used a ratio of 1 family member per mother-child dyad that was reached by any intervention (1 dyad = 1/2 of mothers and children) to calculate numbers. Formative research and baseline survey showed that mothers sought help for CF problems from respondents’ mothers/mothers in law, and family members; subsequently BRAC and other NGOs and government workers held community forums for female family members and women who were pregnant and mothers of young children and engaged them during home visits; additionally, mass media was targeted to male family members and female members of the family were exposed to mass media.

Community leaders were calculated based on monitoring data on community events and mothers’ responses to from whom they seek guidance; we use a ratio of 1/500 dyads reached by any intervention. Formative research and the baseline survey showed that mothers sought help for CF problems from doctors (including the village doctor); community inventions included BRAC and other NGOs conducting community forums and a special newspaper and radio campaign implemented by commercial advertising agencies that was targeted to private and local doctors to raise awareness and fill knowledge gaps on evidence-based IYCF practices and problem-solving.

Religious leaders were based on program design and monitoring records for community events using a ratio of 1 per 500 mother-child dyads reached by any intervention. To build on Koranic teachings that support IYCF, special IYCF materials and sessions were held for religious leaders.

Peers included neighbors, adolescent girls, and visitors from nearby communities, assuming 1 per 4 dyads in reached by any intervention, based on feedback from qualitative studies and media and materials testing that showed these categories of peers shared IYCF information; mass media monitoring showed high to reach to young women 20–40 years.

#### Ethiopia

Health personnel were based on national norms of health provider training; 21,000 facility, outreach and community workers, and volunteers were trained based on a cascade model of training according to training lists. All trainees were drawn from government health services and community health agents approved by government. The numbers in program records were validated by the external project supervisors through observation visits, spot checks, surveys, receipts of training per diems paid, and training materials distributed.

Family members were calculated based on household survey results on family characteristics and mothers’ responses to qualitative research on from whom they seek guidance. We used a ratio of 1 per mother-child dyad reached by any intervention (1 dyad = 1/2 of mothers and children), assuming that at least additional member of the family was engaged as instructed by program staff during each home visit and during health post and health center visits where mothers were accompanied by family members. In addition, families attended community dialogues on IYCF and were exposed to mass media implemented through local audio-visual channels.

Community leaders were calculated based on monitoring data on community events and mothers’ responses to from whom they seek guidance; we used a ratio of 1/1000 dyads reached by any intervention. Formative research and the baseline survey showed that mothers sought help for CF problems from traditional healers and elders. Community inventions involved community dialogues and local media campaigns that were implemented by commercial advertising agencies to reach community-wide audiences.

Religious leaders were calculated based on program design and monitoring records for community events using a ratio of 1 per 500 mother-child dyads reached by any intervention. To build on religious teachings that support IYCF, special IYCF materials and sessions were held for leaders of the Ethiopian Orthodox Church, community-level priests, and lay leaders.

Peers included neighbors, church members, adolescent girls, and visitors from nearby communities, assuming 1 per 4 dyads in reached by any intervention, based on feedback from qualitative studies and media and materials testing that showed these categories of peers shared IYCF information; feedback from local media events showed high to reach to young women 20–40 years.

#### Vietnam

Program records showed that 16,500 health personnel from government health centers, village volunteers, and NGOs managing community support groups were trained. The numbers in program records were validated by the external project supervisors through observation visits, spot checks, surveys of health workers and receipts of training per diems paid, and training materials distributed.

Family members were calculated based on survey results on household characteristics and mothers’ responses to who influences IYCF; we used a ratio of 1 family member per mother-child dyad that was reached by any intervention (1 dyad = 1/2 of mothers and children) to calculate numbers. Formative research and baseline and end-line surveys showed that mothers were influenced by their husbands. The project utilized village volunteers to make home visits and issued invitations to families to bring children for IYCF counseling. Additionally, mass media, supermarket/outdoor displays, internet home pages on IYCF, and competitions for husbands were introduced to engage husbands and family members.

In communities served by Community Support Groups, we calculated 3 facilitators or community leaders per Support Group. This was based on training and supervision reports, materials used for counseling, and monitoring records. No religious leaders were attributed in Vietnam.

Peers were reported to play an important role. We calculated 1 per 2 dyads in Vietnam reached by any intervention.

##### Number of children with improved CF practices

The number of children with improved CF practices was measured through external evaluation studies that calculated incremental changes over time [9–11]. The findings estimated difference-in-differences in key CF practices attributable to the program interventions in Bangladesh (22%) and Vietnam (5.7%), and a pre- and post-intervention adequacy evaluation using regression models and plausibility analyses in Ethiopia (3.5%) [9–11]. We applied the percentages to the population of children 6-23.9 months of age residing in the IPC program areas.

### Calculating unit costs

We used the average annual total cost per year and participants reached per year to estimate the unit costs defined for three metrics: (1) the cost per mother and children reached; (2) the cost per mother, child, and influential individual reached, and (3) the cost per additional child 6 to 23.9 months of age who benefited from MAD, as defined by WHO. We divided the total annual cost by the number of participants reached each year, using the numbers for mother and child reached, the number of mothers, children and influential individuals reached, and number of target children 6-23.9 months old in IPC areas each year with improved practices.

### Sensitivity analysis

We identified the calculation of mothers and children reached as a topic of uncertainty since rigorous evaluations were conducted in a sub-set of IPC program areas where the intensity of program efforts may have been higher than in scaled-up program areas. In the ‘base scenario’ we lowered participant coverage as warranted by the program models in each country. In addition, the percentage of participants reached in each country was further reduced by 15% in all countries and used to calculate alternative unit costs.

## Results

The scale, duration, key stakeholders, and program platforms differed across countries. The same approach to program development and use of multi-intervention strategies involving IPC including community engagement and PE/MM were implemented, but the content, methods of implementation, and behavioral change issues and approaches used were country specific (see Additional File 1).

All programs were effective in improving CF practices according to external impact evaluations by the International Food Policy and Research Institute (IFPRI) [47]. The baseline prevalence of adequate complementary feeding as measured by MAD was 4.8% in Bangladesh, 4.6% in Ethiopia, and 56.4% in Vietnam [9–11]. In Bangladesh, improvement in minimum dietary diversity (MDD) was 16.3% points (pp) and 22.0 pp for MAD. Improvements in timely introduction of foods ranged from 15.5 pp to 28.5 pp. At endline, 63.8% of children in intervention areas achieved MDD and 50.4% achieved MAD [10]. In Ethiopia, improvements in timely introduction of foods, MDD, and MAD were 22.2 pp, 3.3 pp, and 3.5 pp, respectively [9]. In Vietnam, MDD improved by 4.0 pp and MAD by 5.7 pp. The prevalence of MDD and MAD in intervention areas at endline was 90.9% and 81.6%, respectively [11].

### Total and annual incremental financial costs

**Fig. 1:**
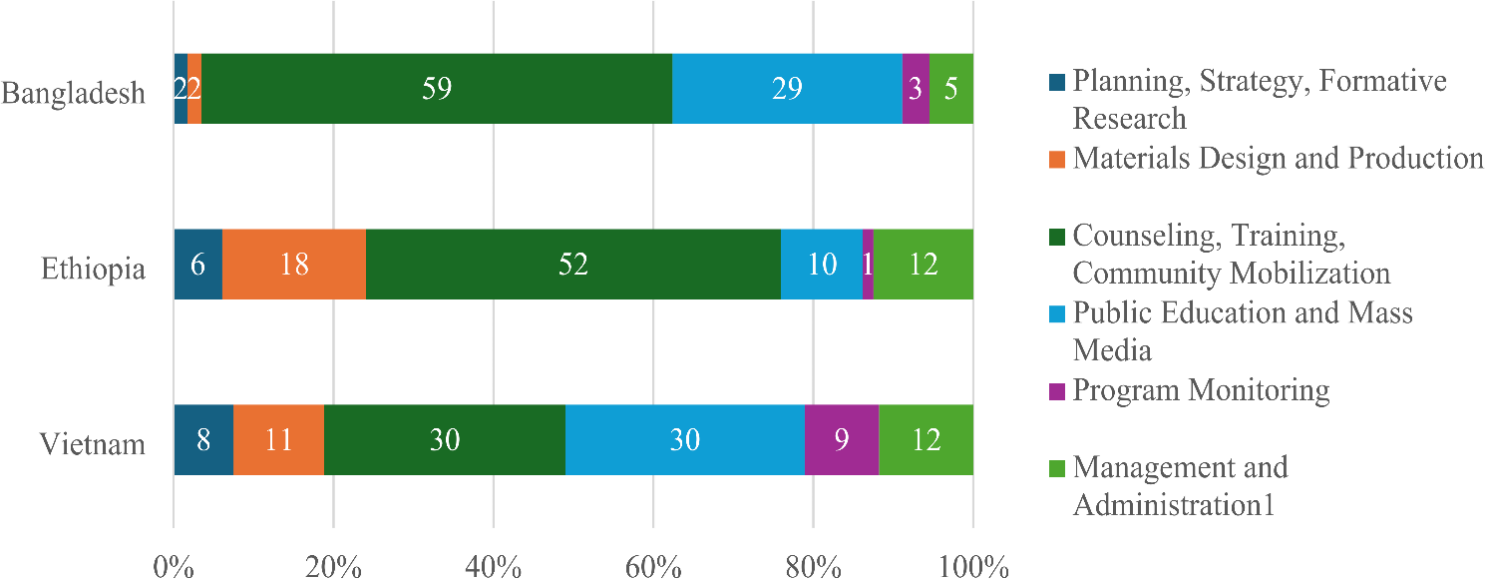
Cost Shares by Program Component (USD 2022). Note: ^1^ Some management & administration costs were loaded on other cost elements and are reflected in other components

The total incremental costs of scaled programs are shown in Table 1. All programs involved differing levels of planning, strategy development and formative research; materials development and production; strengthening health systems to deliver IPC and community-based activities; strategic use of traditional, digital, and social media to reach influential audiences in addition to mothers; monitoring of service delivery coverage and CF practices; and management and administration. While the operational components were the same across countries, the proportion of total incremental costs per component differed (Fig. 1). In Vietnam, almost an equal proportion of total expenditures were incurred for IPC and PE/MM, whereas Bangladesh incurred twice as many resources in IPC as compared with PE/MM, and Ethiopia incurred five times higher expenditures on IPC compared with PE/MM. Program preparatory steps involving strategy development and materials design and testing differed across countries by 4.2 times for strategy and 10.6 times for materials. Strategy costs were low in Bangladesh and could be related to the well-prepared strategy of the NGO BRAC that was already experienced in IPC interventions and required less start-up investment to develop strategy. Ethiopia and Vietnam conducted multiple studies and assessments for designing their strategies, often involving external high-level expertise including willing-to-pay studies for new CF products in Vietnam, and for developing four distinct region-specific program models in Ethiopia. Vietnam developed innovative franchising of IPC counseling services within government health facilities, strengthened infrastructure to make counseling venues client friendly, and used high-quality materials for counseling and training. Expenditures for the IPC component were 1.7 times higher in Bangladesh (including training, home visits and frequent small group education sessions) as compared with Ethiopia and 5.2 times higher as compared with Vietnam. The number of persons trained to deliver quality IPC at scale differed likewise, with over 75,000 trained in Bangladesh, 21,000 in Ethiopia, and 16,500 in Vietnam.

Examples of similar cost structures across CF program components include IPC in Bangladesh and Ethiopia that accounted for 59% and 52% of total expenditures, respectively, and public education/media interventions in Bangladesh and Vietnam that account for 29% and 30% of the total costs, respectively. Expenditures on materials were lowest in Bangladesh at 1.7% and highest in Ethiopia at 18% due to economies of scale in Bangladesh. Monitoring expenditures differed, 1.3% in Ethiopia to 9.3% in Vietnam. It is unclear whether monitoring investments were inadequate or more efficient in Ethiopia; Vietnam developed a sophisticated system to track new and returning clients at each facility to document the franchise model innovation, modified activities based on monitoring data, and awarded incentives for high performing to facilities based on monitoring data. Across the three countries, greater costs were incurred on monitoring than is reflected here as these costs were embedded in the payments to implementing partners for IPC and PE/MM. Administrative and management expenditures comprised 5.5% in Bangladesh, 11.8% in Ethiopia, and 12.5% in Vietnam. The latter two countries utilized more international partners as compared with Bangladesh where national expertise was adequate in key areas of implementation.

**Table 1:** Total and annual costs of complementary feeding programs by country (2022 USD)

Program Component	Bangladesh	Ethiopia	Vietnam
**Incremental program costs**			
Planning, Strategy, Formative Research	184,286	437,341	295,547
Materials Design and Production	179,787	1,294,340	444,642
Counseling, Training, Community Mobilization	6,188,236	3,736,014	1,185,554
Public Education and Mass Media	3,014,636	735,890	1,175,091
Program Monitoring	357,191	95,283	366,525
Management and Administration^1^	575,339	899,152	462,345
Total Program Costs	10,499,475	7,198,020	3,929,704
**Duration**			
Total program duration (period of exposure)	4.7 years (46 months)	4.0 years (30 months)	4.1 years (36 months)
Annual Program Costs	2,233,931	1,799,505	958,464

Note: ^1^ Some management and administration costs were loaded onto other cost elements and are reflected in the rows above

### Participants reached

#### Mothers and children (6-23.9 months)

The maximum number of mothers and children in the 6–23.9-month age reached through either IPC or PE/MM was 2.49 million in Bangladesh, 1.12 million in Ethiopia, and 1.02 million in Vietnam (Table 2). The program platforms that reached maximum scale were IPC in Bangladesh and Ethiopia and PE/MM in Vietnam. This reflects high coverage of community-based health workers and volunteers in Bangladesh, health extension workers based in health posts in Ethiopia, and media habits of mothers in Vietnam.

#### Other key participants

Five categories of influential persons were reached in addition to mothers and children reached by IPC and/or PE/MM (Table 3). Influentials were needed to support mothers in practicing the recommended CF and included members of the family (e.g., father of the child, elders, mothers in law), health care workers, community leaders including ‘local doctors’, peers, and religious leaders. The programs incurred costs for identifying the specific categories, developing materials for each, and engaging various platforms and media channels to deliver them. For example, Vietnam reached fathers through digital and social media campaigns, and Bangladesh conducted a special media campaign to reach local doctors.

#### Children benefited

This was measured by external evaluations in a representative sample of children in IPC implementation areas [9–11]. Improved practices, specifically the MAD indicator includes a combination of meal frequency and dietary diversity and indicates both micronutrient adequacy and the quantity of food consumed during the previous day or night. Both the size of the child population and magnitude of improvements in CF differed by country (Table 4).

**Table 2:** Mothers and children reached annually through program interventions

Variable	Bangladesh^1^	Ethiopia^2^	Vietnam-franchise^3^	Vietnam-CSG^3^
**High intensity IPC and PE/MM areas**				
Numbers of mothers & children residing in high intensity areas	692,935	893,176	1,011,429	60,000
Percentage reached by IPC	92%	38%	42%	50%
Number reached by IPC	637,500	339,407	450,000	30,000
Percentage reached by PE/MM	73%	30%	36%	36%
Number reached by PE/MM	505,842	267,953	385,714	21,600
**Low intensity IPC and PE/MM areas**				
Numbers of mothers & children residing in low intensity areas	2,508,424	2,067,351	3,315,342^a^	na
Percentage reached by IPC	74%	38%	0%	na
Number reached by IPC	1,856,234	785,593	0	na
Percentage reached by PE/MM	67%	24%	31%	na
Number reached by PE/MM	1,680,644	496,164	1,027,756	0
**All IPC and PE/MM areas**				
Mothers & children residing in IPC areas	3,201,359	2,960,527	1,071,429	
Mothers & children residing in areas receiving IPC and/or PE/MM	3,201,359	2,960,527	4,386,771	
Mothers & children reached by IPC^b^	2,493,734	1,125,000	480,000	
Mothers & children reached by PE/MM^c^	2,186,486	764,117	1,027,756	
Maximum reached by any intervention	2,493,734	1,125,000	1,027,756	

Indicators: ^a^ Only MM was implemented in low intensity program areas in Vietnam; no IPC here. ^b^ with/without PE/MM in all countries. ^c^ with/without IPC in all countries.

Numbers reached: ^1^ Bangladesh: Calculated from 1.7 million mothers of children < 2 years reached in 50 upazilas over 2 years [41, 48], 92% IPC, 73% and 67% PE/MM coverage [10]; 74% is the discounted (intensive − 20%) IPC coverage in scaled up areas based on comparing the intensive and scaled up models [42]. ^2^Ethiopia: Calculated from 1.5 million mothers of children < 2 years reached in 295 woredas over 2 years [41, 43], 38% IPC, 30% PE/MM coverage in 89 intensive areas [9] and 40% of intensive coverage in low intensity areas based on process evaluation [44]. ^3^Vietnam: Calculated from 2.3 million mothers of children < 2 years reached nationally over 3 years [45], catchment area population of CHCs [49], 42% IPC (franchise) and 50% (CSG), 36% PE/MM coverage in intense and 31% PE/MM coverage in low intensity areas [11], and 30,000 mothers and children 6-23.9.9 months reached through community support groups for IPC [46] at an assumed coverage of 50%.

Acronyms: CSG = community support group, IPC = interpersonal communication, MM = mass media, na = data not available, PE = public education

**Table 3:** Total participants reached in Bangladesh, Ethiopia, and Vietnam

Mothers, Children, and Influential Persons Reached			
Mothers and Children	Bangladesh	Ethiopia	Vietnam
Maximum number of mothers and children reached by any method	2,493,734	1,125,000	1,027,756
Mother/child dyads reached by any method (dyad = 1/2 mothers and children)	1,246,867	562,500	513,878
**Influential Persons**			
i) Health personnel (program records)	75,000	21,000	16,500
ii) Family Members, assuming 1 per mother-child dyad reached by any intervention (1 dyad = 1/2 of mothers and children)	1,246,867	562,500	513,878
iii) Community Leaders, assuming 1/500 dyads reached by any intervention in Bangladesh, 1/1000 in Ethiopia, 3 per Support Group in Vietnam	2,494	563	801
iv) Religious Leaders, assuming 1 per 500 mother-child dyads reached by any intervention in Bangladesh and Ethiopia	2,494	1125	-
v) Peers, assuming 1 per 4 dyads in Bangladesh and Ethiopia; 1 per 2 dyads in Vietnam reached by any intervention	311,717	140,625	256,939
Total number of influential persons reached, sum of i) through v) above	1,638,572	725,813	788,118
**Total Participants Reached Including Mothers**,** Children**,** and Influential Persons**	4,132,306	1,850,813	1,815,874

Note: Influential persons are calculated based on formative studies and program descriptions of interventions designed to reach specific categories of persons reported by mothers to support recommended CF practices

**Table 4:** Children Benefited^1^

Variable	Bangladesh	Ethiopia	Vietnam
Children with improved CF practices, Minimum Acceptable Diet	22%	3.5%	5.7%
References for RCTs	(10)	(9)	(11)
Mothers and children in IPC intervention areas (from Table 2)	3,201,359	2,960,527	1,071,429
Children in IPC intervention areas (1/2 mothers and children)	1,600,680	1,480,264	535,715
Number of children with improved CF^1^	352,149	51,809	30,536

^**1**^ Minimum Acceptable Diet as defined by WHO [47]

### Unit costs per participant reached and per child benefited

The cost per participant reached by either IPC and/or PE/MM differed 1.8 times across countries and was calculated by dividing the annual costs by the number of participants reached each year (Table 5). When adding all categories of participants needed to achieve impacts, the cost per participant reduced to $0.50-$1.00. Although using different program platforms to reach scale, Bangladesh and Vietnam were similar in cost-effectiveness both in reaching mothers and children (approximately $ 0.90 per person) and in reaching all participants ($ 0.53 per person). There was a 5.5-fold difference in the cost per child improved across countries.

**Table 5:** Unit costs of complementary feeding programs (2022, USD)

Variable	Bangladesh	Ethiopia	Vietnam
**Program costs (from Table ** 1 **)**			
Total program costs (USD)	$ 10, 499,465	$ 7,198,020	$ 3,929,704
Annual program costs (USD)	$ 2,233,929	$ 1,799,505	$ 958,464
**Participants reached in program areas annually**			
Mothers and children reached by at least one intervention (Table 2)	2,493,734	1,125,000	1,027,756
Total number of participants (mothers, children, influentials) (Table 3)	4,132,306	1,850,813	1,815,874
**Number of children benefited in program areas annually (from** Table 4**)**			
Children with improved CF practices (Minimum Acceptable Diets)	352,149	51,809	30,536
**Unit costs per participant reached and child benefited (USD annual)**			
Cost per mother and child reached	$ 0.90	$ 1.60	$ 0.93
Cost per all participants reached	$ 0.54	$ 0.97	$ 0.53
Cost per child with improved CF practices	$ 6.34	$ 34.73	$ 31.39

### Sensitivity analysis

As shown in Table 6, accounting for uncertainties in country program coverage by reducing the population reached by 15% resulted in increased costs per mother and child. Similarly, the cost per all participants reached increased among the countries. The implications for budgeting are that countries need to invest $1 to $2 per mother and child they aim to reach with CF interventions on a large scale.

**Table 6:** Sensitivity analysis: 15% reduction in participants reached

Participants Reached	Bangladesh	Ethiopia	Vietnam
**Number of participants**			
**Base scenario**			
Mothers and children reached by any intervention (from Table 2)	2,493,734	1,125,000	1,027,756
Mothers, children and influentials reached (from Table 3)	4,132,306	1,850,813	1,815,874
**Alternate scenario: 15% lower reach**			
Mothers and children reached by any intervention (from Table 2)	2,119,674	956,250	873,593
Mothers, children and influentials reached (from Table 3)	3,512,460	1,573,191	1,543,493
**Program costs (USD)**			
Annual program costs (from Table 1)	$ 2,233,929	$ 1,799,505	$ 958,464
**Unit costs of participants reached (USD)**			
**Base scenario**			
Cost per mother/child reached	$ 0.90	$1.60	$ 0.93
Cost per mother/child/influential reached	$ 0.54	$ 0.97	$ 0.53
**Alternate scenario: 15% lower reach**			
Cost per mother/child reached	$ 1.05	$ 1.88	$ 1.10
Cost per mother/child/influential reached	$ 0.64	$ 1.14	$ 0.62

The results suggest that programs for improving child nutrition can reach large scale rapidly through interventions implemented by strengthening and integration of IYCF into existing large-scale delivery systems. In programs included in this study, scale was at almost national levels in Bangladesh and Vietnam and two-thirds of the national population in Ethiopia. The program models were effective in improving CF practices within a 4-year period according to external impact evaluations, although some attenuation of coverage and impact appears to have occurred during expansion that took place through new delivery systems and diverse cadres of workers. While the operational components used to achieve impact at scale were the same across countries, the proportion of costs per component differed. In Vietnam, almost an equal proportion of total expenditures were incurred for IPC and PE/MM, whereas Bangladesh incurred twice as many resources in IPC as compared with PE/MM, and Ethiopia incurred five times higher expenditures on IPC compared with PE/MM. Program preparatory steps involving strategy development and materials design and testing differed across countries by 4.2 times for strategy and 10.6 times for materials, reflecting program needs and local prices. The maximum number of mothers and children was 2.49 million in Bangladesh, 1.12 million in Ethiopia, and 1.02 million in Vietnam. The program platforms that reached maximum scale were IPC in Bangladesh and Ethiopia and PE/MM in Vietnam. This reflects high coverage of community-based health workers and volunteers in Bangladesh, health extension workers based in health posts in Ethiopia, and media habits of mothers in Vietnam. Each country needs to identify and leverage its delivery strategies and invest the resources needed for strengthening capacity to achieve results. When strategically designed and implemented with rigorous monitoring, countries can achieve impacts at a cost of $1 to $2 per mother and child reached.

## Discussion

The aim of this analysis was to document the financial cost of improving child nutrition. According to WHO 45 million children globally were estimated to be wasted (too thin for their height) in 2022, and nearly half of deaths among children under 5 years of age are attributable to undernutrition [50]. Large gaps have been documented in feeding age-appropriate amounts of nutrient-dense foods to children during 6 to 23.9 months of age leading to a spiral of malnutrition and infection [3]. Preventive interventions can deliver improvements in knowledge and provide support to mothers leading to improved CF practices in diverse LMICs [9–11, 51]. Despite progress in generating evidence on the effectiveness of CF programs, important gaps remained in understanding how to budget for national programs. This study offers evidence of incremental financial costs and costs per participant incurred for designing and delivering locally adapted, context-specific interventions at scale.

In Bangladesh and Ethiopia, IPC incurred the greatest expenditures while in Vietnam PE/MM was equally resourced as IPC. Bangladesh had sufficient funding, reached scale, and achieved adequate quality and intensity in IPC and PE/MM, and this combination improved CF practices and shifted CF norms that continued to influence IYCF practices even after funding stopped [52, 53]. Ethiopia did not have sufficient financial and human resources to achieve quality and intensity at community level and would have needed greater strengthening of existing health systems and links with food system interventions to address food insecurity [44]. Vietnam delivered an innovative high-quality service delivery model that was franchised through government health centers, but mothers with children in the CF age group did not utilize health facilities sufficiently once their child’s immunization schedule was completed [46, 54]. Mothers in Vietnam returned to work after their child reached four months; extended maternity accommodations and reaching mothers through digital tools can be important ways to close this gap in future programs. Early detection and remediation of weaknesses in program design and implementation is an important lesson learned.

Use of mass media in Vietnam was highly effective in achieving large coverage of mothers and children and other influentials, but IPC contacts were also needed to achieve improved practices [11, 55]. In Bangladesh, integrating IPC into existing service delivery systems of the national NGO BRAC that was reaching mothers repeatedly through home visits with a broader health package helped to reduce initial startup costs. In Ethiopia and Vietnam, greater investments in improving service delivery platforms would have been needed to achieve the desired program reach and intensity. Both Bangladesh and Vietnam benefited from well-developed mass media sectors to achieve scale whereas Ethiopia needed to rely more on mobilizing frontline health workers who had a weaker chain of cascading capacity and materials to volunteer networks at community level [44]. Although Ethiopia gave high priority to CF, the program faced serious logistical challenges to reach their target population and had limited options for investing in PE/MM, investing a higher proportion of costs for IPC. Additionally, low levels of CF practices were considered a crisis in Bangladesh and Ethiopia and linked to high levels of child undernutrition; higher total investments were made for improving CF in Ethiopia and Bangladesh where total costs for CF programs were $ 7.2 million and $ 10.5 million respectively as compared with $ 3.9 million in Vietnam. Achieving CF improvements on a large scale was the primary objective in all three countries, and the program investments resulted in a range of positive outcomes at scale.

The results illustrate considerable benefits of integrating CF interventions into existing services and obtaining economies of scale. In Vietnam, the average unit cost was $ 0.93 per mother and child reached, $ 0.90 in Bangladesh, and $ 1.60 in Ethiopia. Both Vietnam and Bangladesh’s strategy and investments were moderately successful in reaching scale at a unit cost of less than one US dollar, and Bangladesh obtained a 22% increase in the prevalence of adequate CF practices (Menon et al. 2016). In Ethiopia, the average cost per mother and child reached was higher and IPC logistical difficulties and low household food security limited their achievement in improved CF, obtaining an 3.5% change in minimum acceptable CF practices [9]. Vietnam invested almost equally in both IPC and mass media, achieving less than one $ in unit costs per mother and child reached, but their heavy dependence on facility visits, with less investment in delivering CF counseling directly to mothers through other channels, limited their scale and impact, yielding an increase of 5.7% in adequate CF practices in the general population and 8% increase in mothers with at least one IPC contact [11]. Mothers/caregivers and children in Vietnam already had several fold higher CF indicators at baseline as compared with Bangladesh and Ethiopia and may have had reduced potential to achieve higher impacts.

Program scale in our study reached one to 2.5 million mothers and children each year per country over multiple years, not through modelling but actual implementation. The study suggests that the situational diagnostics, ongoing remediation during scaling up through responding to monitoring, and flexibility are required; this implies a minimal level of investment as seen in the more than 6-fold difference in improved CF prevalence achieved across countries, with higher investments resulting in larger scale and greater improvements in CF outcomes.

We are unable to separate and calculate the cost of participants reached through IPC or PE/MM independently of one another because the PE/MM was used to drive IPC reach by promoting service utilization through mass media [11]. Evidence shows that IPC providers were more motivated and knowledgeable when exposed to mass media and this translated into better IPC results [52]. The costs represent increment and not the total cost of the programs; our aim is to fill gaps in budget planning for countries to make further improvements to achieve behavior change on a large scale by mainstreaming the additional interventions into existing program platforms. Also, we do not have measures of efficacy for behavioral outcomes in the scaled-up areas and therefore based on descriptive information and program monitoring feedback, we applied sensitivity analysis to produce the most likely ranges of unit costs. Other limitations include the lack of input-level cost data and inability to provide more disaggregated activity-based costs. We do not have data for attributing results to only IPC or only PE/MM. Nevertheless, the main components of scaled-up CF programs were costed and provide illustrative information for planning future programs. Finally, we used the base population of only IPC-designated areas in each country as denominators for the number of children benefited. The results represent conservative estimates and actual unit costs may have been lower if non-IPC area impacts were included and if less stringent CF indicators such as MMF alone or MAD alone or the age at introduction of foods were considered instead of the MAD indicator.

## Conclusion

To aid planning and budgeting, this study provided the costs of large-scale programs with differing combinations of country-specific delivery channels including granular details on strategies and their financial costs. Expanded delivery of child health services and increasing utilization of health centers by mothers, combined with rising availability of PE/MM platforms, offer renewed possibilities for delivering large-scale improvements in CF practices to prevent malnutrition in countries struggling to reduce elevated levels of child malnutrition through improving diets of children. The implications of not acting on these opportunities are escalated costs of treatment for severe malnutrition and continued high mortality and cognitive limitations among undernourished children. Global guidance and past publications advocate for using behavioral science principles to address barriers to adequate CF at multiple levels and utilizing existing high-reach platforms to achieve improvements in CF practices on a large scale [39, 41], but costs of these initiatives were not known. This study documented the costs disaggregated by essential CF program components in diverse LMICs and illustrated financial implications for similar countries. The novelty of this cost study is that interventions were implemented at scale in LMICs and produced substantial impacts on CF as documented through rigorous external evaluations. The results deepen our understanding of what it takes to prevent child malnutrition and to budget and forecast adequate resources to prevent child malnutrition.

## Electronic supplementary material

Below is the link to the electronic supplementary material.

**Additional File 1**: Overview of complementary feeding program characteristics, interventions, and impacts.

**Additional File 2**: Overview of data sources on program participants and ‘influential persons’ reached by interventions in countries.

## Data Availability

Expenditure data are provided in the tables within the manuscript. All datasets for the coverage data have been anonymized and are publicly available on the Harvard Dataverse repository at https://dataverse.harvard.edu/dataverse/IFPRI and A&T’s repository on Harvard Dataverse.
