# Supplementary material for: Incremental financial costs of strengthening large-scale child nutrition programs in Bangladesh, Ethiopia, and Vietnam: retrospective expenditure analysis
Source: Global Health. 2025 Apr 21;21:21. doi: 10.1186/s12992-025-01118-3 (PMC12013185; doi:10.1186/s12992-025-01118-3)
Supplement: Supplementary file 1 — Additional File 1: Overview of complementary feeding program characteristics, interventions, and impacts. [file 12992_2025_1118_MOESM1_ESM.docx]

**Additional File 1.** **Overview of complementary feeding program characteristics, interventions, and impacts**

| **Characteristics** | **Bangladesh** | **Ethiopia** | **Vietnam** |
| --- | --- | --- | --- |
| Scale and locations | Nationally scaled up through IPC and mass media in 456/490 upazilas; 231 of these are IPC upazilas of which 50 are intensive | Scaled up in Amhara, Oromia, SNNP, Tigray regions mainly through IPC in 295/523 woredas; 89 of these are intensive IPC woredas | Nationally scaled up mainly through mass media; IPC in 121 franchises at provincial and district levels and 660 franchises at the commune level, IYCF support groups in 267 villages. |
| Key stakeholders | MOH policy and technical guidelines, UNICEF, national NGO (BRAC) service delivery (IPC), private sector media; national IYCF alliance. | MOH policy and service delivery HEP, IFHP (World Vision), UNICEF, 4 regional NGOs; Women’s Associations, FBOs; social marketing agency public education. | National Institute of Nutrition (MOH), UNICEF, provincial health departments (IPC, CM), National Women’s Association, Save the Children, private sector media agencies. |
| Activity timelines | 2010-2011: Formative research, initial model specified, and pilot tested in BRAC health program platforms, materials developed distributed, basic training. 2010-2014: Phased scale up of IPC, public education/media start 2010.  2011-2014: Baseline and endline evaluation surveys (A&T 2017). | 2010-2011: Formative research, message content and region-specific models developed and pilot-tested, materials developed distributed, basic training. 2010-2014: Phased scale up of IPC, public education/ media start 2011. 2011-2014: Baseline and endline evaluation surveys (A&T 2017). | 2010-2011: Formative research, franchise model designed, materials developed, government agreements.  2010-2014: Training, coaching, and supportive supervision; franchises, support groups scaled up, public education/media start in 2010.  2011-2014: Baseline, endline evaluation surveys (A&T 2017). |
| Durations | 46 months of intervention exposure (4.7 years total including planning and dissemination). | 30 months of intervention exposure (4 years total including planning and dissemination). | 36 months of intervention exposure (4.1 years total including planning and dissemination). |
| **INTERVENTIONS** | | | |
| Interpersonal  Communication (IPC):  a. Individual and small group counseling of mothers  b. Community level | a) Home-visits by 2 cadres (SS, PK) of BRAC health workers and volunteers; group counseling at community level by 1 cadre (SK).  Counseling coverage: 81% mothers were visited and counseled on CF.  b) Field managers facilitated multiple community events in IPC areas for 9 categories of influential audiences (e.g., teachers, private doctors, Imams). Audience-specific tools. (AliveThrive 2015; Menon et al. 2016; Sanghvi et al. 2016) | a) Counseling on CF at health posts (23% mothers) and home visits (26% mothers) by HEW. 60.7% mothers recalled CF messages in IPC tools. Counseling coverage: 45%  b) Food demonstrations and group discussions at village gatherings in IPC areas to address preparation and feeding of enriched CF; by HEW, Women’s/Health Dev. Armies (WDAs/HDAs). (A&T 2012; A&T 2015a; Kim et al. 2016) | a) Facility-based counseling through 781 franchises in 15/63 provinces.  Counseling coverage: 42% of mothers visited counseling franchises.  b) VHWs encouraged mothers to seek counseling at facilities in franchise catchment areas; 76 community support groups established with 2 trained facilitators per support group in hard-to-reach ethnic minority areas with a district or provincial franchise. (A&T 2015b; Nguyen et al. 2014; Rawat et al. 2017) |
| Public education/mass media (PE/MM) | 4 TV and 4 radio spots on CF through commercial channels; 1 animated film for government broadcasts; multi-year campaigns; shows in media-dark areas; doctors reached through newspapers and radio. Coverage: 74% recall of CF messages, lower in non-IPC areas. (Menon et al. 2016) | 7 radio and 7 TV spots in 3 languages; 4-episode radio drama, music videos, and 45-minute drama for TV and mobile vans; episodes broadcast through 8 media houses. Several messages on CF introduction, enriching porridge, dietary diversity for CF. Coverage: 31% recall of CF messages. (Kim et al. 2016) | 1 TV spot on CF on dietary diversity/iron-rich foods; print materials, billboards, screens in supermarkets and hospitals, bus ads, digital materials, Webpage, Facebook, TV spots, mobile phone app., national and provincial channels. Coverage: 36% recall of CF messages, lower in non-IPC areas. (Rawat et al. 2017) |
| **IMPACT ON CF PRACTICES** | | | |
| Dietary diversity (MDD), number of meals (MMF), both (MAD) | Increased by 16.3%*for MDD, 14.7%** for MMF, 22.0% *** for MAD, as compared to control areas. (Menon et al. 2016) | Increased by 3.3%* for MDD, 26.2%*** for MMF, 3.5% * for MAD, as compared to control areas. (Kim et al. 2016) | Increased by 6.4%* for MDD, 3.2% for MMF, 5.7 for MAD overall and 8%* in mothers with > 1 IPC, as compared to control. (Rawat et al. 2017) |
| Dose response relationships with more contacts and more channels | Compared with one intervention, IPC + public education/media increased the odds of MDD from 1.1 to 2.8, MMF 1.3 to 3.6, MAD 1.0 to 3.8. (Menon et al. 2016) | Greater number of home visits meant increased odds of MDD 1.8–4.4, MMF, and MAD; heard more radio spots had 3 times greater odds of MDD and MAD. (Kim et al. 2016) | MDD and MAD improved more in intervention areas where public education/media was present among mothers with > 1 IPC visit to a franchise (Rawat et al. 2017) |

*p < 0.05, **p < 0.01, ***p < 0.001. CF=complementary feeding, DDE: difference in difference estimate, FBO=faith-based organization, FLW=frontline worker, HDA= health development army, HEP=health extension program, HEW=health extension worker, IFHP= Integrated Family Health Program, IPC=interpersonal communication. MAD= minimum acceptable diet (both MDD+MMF), MDD=minimum dietary diversity, MM=mass media, MMF=minimum meal frequency, MOH= ministry of health, PE=public education, PK=*pushti kormi (nutrition worker)*, SK=*shasthya kormi* (health worker), SNNPR= Southern Nations, Nationalities and Peoples’ Region, SS=*shasthya sebika* (community health volunteer), VHW=village health worker, WDA= women’s development army.

**Notes on Table 1**

The programs conducted IPC through home visits, outreach from facilities, community health education groups, community events and networks, and/or one-on-one or group counselling during health facility visits and aimed to build knowledge, skills, self-efficacy, and belief in the benefits of recommended CF practices. All countries engaged PE/MM to reach multiple categories of influential persons at scale including fathers and other family decision-makers, community opinion leaders, health workers and ‘doctors’, program managers, and policy makers; the aim was to shift norms and remind and motivate mothers/caregivers and frontline workers about what desirable CF practices are, why they are critically important, and how to achieve adequacy by prioritizing CF within their household budgets. The PE/MM component was integral to achieving IPC at scale and designed to increase the demand for IPC, volume of CF visits at facilities and in the communities and build the credibility of community workers making home visits. Program durations including planning, research, strategy design, media and materials development, and delivery and monitoring ranged from 4 years in Ethiopia to 4.1 years in Vietnam and 4.7 years in Bangladesh.

Three cadres of NGO workers counseled mothers in Bangladesh during early implementation. Later thousands of frontline workers from government and more NGOs were scaled up through a stakeholder consortium created for this purpose; over 75,000 were trained inclusive of providers, counselors, volunteers, facilitators, supervisors, and managers. In Bangladesh, dietary diversity and reduction of unhealthy foods were the focus; activities were conducted at community level through home visits and small groups, dialogue with the broader community of opinion leaders, local doctors, peers, extended family members. Radio and TV were used to illustrate recommended CF practices, particularly diverse sources (animal source foods, pulses), frequency of feeding, how to address perceived ‘poor appetite’, handwashing, and discouragement of unhealthy foods, and included a ‘doctor’s campaign’ for formal and informal medical providers (AliveThrive 2015).

In Ethiopia, the age of introduction, use of multiple foods, and appropriate consistency and quantity were the special focus of CF practices. Several platforms were used, e.g., government cadres of health extension workers and volunteers for counseling and community mobilization, about 21,000 in all including supervisors, trainers, and managers. Health extension workers and volunteers at community level conducted home visits and group meetings (A&T 2015). Private agencies designed and implemented local media and mass media, e.g., radio programs and music videos broadcast through regional radio stations, loudspeakers, and vans to obtain broader social support for recommended CF practices.

The Vietnam program focused on age-appropriate feeding practices, iron-rich foods, and continued breastfeeding and used facilities for IPC, establishing a franchise model for counseling services in government health facilities at commune/sub-district, district, and provincial levels. Village health workers made visits to home of children under 2 to motivate mothers for attending counseling sessions at facilities (A&T 2013). In addition, community IYCF support groups were established in areas with low access to commune, district, and provincial level franchises (Nguyen et al. 2021). They trained an estimated 16,500 health personnel and community facilitators and engaged distinct types of commercial mass media and digital channels for public education and maternal reinforcement of CF practices and use of counseling services.

**METHODOLOGY – Explanatory Notes**

*Estimating incremental financial costs*

We organized costs under categories that aligned with the specific program components of CF programs. Most activities were clearly identifiable as planning, design, development; materials production; delivery of IPC involving in-person dialog with mothers or influential persons; delivery of public education/media; and monitoring. We combined expenditures on individual counseling and community mobilization activities with training under IPC; these activities were implemented together, jointly invoiced, and paid to the same implementing agencies. Mass media activities were stand-alone, and included the development of digital, radio and TV content, and dissemination through commercial advertising agencies. Examples include placement of content in radio, TV, social and digital media channels; media purchases (e.g., advertising time slots in radio and TV programs); printing and placement of printed materials on bus-wraps, billboards and display areas around community centers and health facilities; and media monitoring.

The cost of management and administration of program activities was incurred in part by implementing agencies, invoiced together, and embedded within activity costs. Additional administrative and management costs were incurred by Alive & Thrive and documented separately. The total costs were mutually exclusive and exhaustive of all CF program activities. Since CF activities were implemented through child health services and focused on children in the 6–23-month age group, we excluded costs incurred for exclusive breastfeeding promotion through antenatal care and delivery and postpartum services that involved pregnant and nursing women with newborns and young infants.

*Calculation of the number of participants*

Evaluation sub-areas were selected by country teams in consultation with IFPRI for providing the best representation of the scaled-up programs. Program areas for scaling up the interventions were jointly selected by government, implementing agencies, and Alive & Thrive. Numbers of mothers and children in the 6-23-month age-group residing in program areas were obtained from program monitoring data, 2012-2014 national and regional population data, program records, and the following World Bank population databases: https://data.worldbank.org/indicator/SP.POP.TOTL. The program approach for maximizing sustainable scale was to strengthen IPC interventions in existing service delivery platforms where infrastructure existed for repeated contacts between service providers and mothers of children in the 6–23-month age group. Mass media, including radio and TV in Bangladesh and mass, digital, and social media in Vietnam were assessed by program teams to have almost national coverage. Ethiopia used radio and social promotion platforms in program regions. Based on monitoring data, and health department population figures for women and children in sub-regions and catchment areas reached by the programs, in Bangladesh 1.7 million mothers and children below 2 years were reached in 50 upazilas during two years, in Ethiopia 1.5 million mothers of children below 2 years were reached over two years, and in Vietnam 2.3 million mothers of children under the age of 2 were reached through counselling at health facilities and home visits, community groups, and public education using local media, social media, and mass media (Frongillo 2020; WHO 2016).

*Number of mothers and children reached:* Mothers of children 6-23 months of age in randomly selected households were interviewed by evaluators in program areas and non-program through endline evaluation surveys. In Bangladesh, for IPC, 92% mothers reported visits by frontline workers (FLWs) to discuss CF; for public education/media, 73% in IPC areas and 67% in non-IPC areas heard and recalled CF messages from TV spots (Menon et al. 2016). In Ethiopia, IPC coverage was calculated at 45% from reported home visits by health extension workers or by a volunteer or mothers’ visits to a health post where CF was discussed; for public education/media in Ethiopia, 30% of mothers heard and recalled CF messages from radio spots at endline (Kim et al. 2016). In Vietnam, for IPC, 42% of mothers reported visits to the franchise, while for public education/media, 36% and 31% of mothers in IPC and non IPC areas respectively recalled messages from TV spots on CF (Rawat et al. 2017). The coverage for non-evaluation areas (low intensity) areas was based on process assessments and adapted models used in scale up (non-evaluation or low intensity) areas and the reported numbers of mothers and children reached in non-intervention areas (BRAC 2014; Frongillo 2020; Kim et al. 2015).

*Number of influential persons reached:* The programs applied the Socio Ecological Model that requires programs to reach relevant categories of influential individuals and engage them in reducing barriers faced by mothers in following recommended CF practices. Investing in these audiences is as critical as investments in counseling of mothers; influentials can protect the investments made in counseling mothers by facilitating mothers’ environments to practice recommended CF in the near and long terms. They include key members of the family and community with influence over social norms, control over procurement and access to household food resources, and/or influence over accessing health services and community events where mothers could receive CF counseling and engage in dialogue. Specific channels used to reach influentials were identified by program teams through media habits surveys and formative studies, e.g., radio and newspapers to reach doctors in Bangladesh and social media and websites to reach fathers in Vietnam. Based on our knowledge of community dynamics, we estimated social leaders in program areas at one per five hundred mothers reached (Bangladesh), one per 1,000 households (Kebele) in Ethiopia, and three per Community Support Group in Vietnam. Religious leaders in IPC areas were actively engaged by the Bangladesh and Ethiopia programs and calculated at 1 per five hundred mothers reached in Bangladesh and Ethiopia. We calculated peers at one per four mothers reached for Bangladesh and Ethiopia, and one per two mothers for Vietnam. The sum of these five groups yields the total number of influential persons reached.
