# Supplementary material for: Incremental financial costs of strengthening large-scale child nutrition programs in Bangladesh, Ethiopia, and Vietnam: retrospective expenditure analysis
Source: Global Health. 2025 Apr 21;21:21. doi: 10.1186/s12992-025-01118-3 (PMC12013185; doi:10.1186/s12992-025-01118-3)
Supplement: Supplementary file 2 — Additional File 2: Overview of data sources on program participants and ‘influential persons’ reached by interventions in countries. [file 12992_2025_1118_MOESM2_ESM.docx]

**Additional File 2: Overview of data sources on program participants and ‘influential persons’ reached by interventions in countries**

| **Country, author, year, title** | **Study design, data sources** | **Findings on participants and interventions** | **Findings on categories of influentials and interventions** |
| --- | --- | --- | --- |
| **Bangladesh** | | | |
| Menon et al, 2016. Combining Intensive Counseling by FLWs with a Nationwide Mass Media Campaign Has Large Differential Impacts on CF Practices but Not on Child Growth | External cluster randomized evaluation of IYCF practices and coverage using cross-sectional surveys at baseline and endline 4 years later. J Nutr. 2016;146 (10):2075-84. | 92% of mothers were reached through counseling; 73% were reached through mass media in the areas that also received; 67% were reached through mass media in control areas indicating the national mass media reach. | NA |
| BRAC, 2014. Scaling Up and Sustaining Support for Improved Infant and Young Child Feeding | Monitoring and implementation databases document phases of model development, replication and adaptation of the model in multiple national programs and platforms. | Compared to 92 percent of mothers who received counseling or IPC on IYCF from a program worker, a reduction of 20% in coverage was assumed as the program expanded to new areas in subsequent phases. The model was streamlined based on initial findings and key elements incorporated in BRAC’s existing national Essential Health Care (an additional 140 rural subdistricts and 9 city corporations) and 82 MNCH program subdistricts. BRAC trained government staff who additionally expanded coverage through national maternal and child health services in urban and rural areas. | Home visits for IPC included dialogue with older women and fathers of children. Pregnant women and mothers of children below 2 years received monthly groups education and discussion sessions. IYCF education sessions were integrated into ongoing multiple community-based women’s groups per subdistrict and social mobilization forums on IYCF for opinion leaders e.g. village and town doctors, local government officials, religious leaders, fathers of young children, adolescents, and religious leaders. |
| Nguyen at al, 2019. Intervention Design Elements Are Associated with Frontline Health Workers’ Performance to Deliver Infant and Young Child Nutrition Services in Bangladesh and Vietnam | Survey data from 2010 and 2014 used for multiple regression to test differences in FLW exposure and performance outcomes; path analyses to track exposure links to performance outcomes in FLW and end-users. Curr Dev Nutr 2019;3:nzz070. | NA | In total, an estimated >75,000 health workers, managers, and community-based development agents throughout the country were trained to deliver IYCF counseling or IPC. Additionally, mass media reached a number of influentials; evaluation of health workers exposed to mass media showed improved performance scores. |
| BRAC, 2011-2014. Quarterly Training and Community Events Delivery Reports | Implementation reports submitted quarterly on the number of training sessions completed and attendees per session, validated through spot checks, observation visits and expenditure receipts | NA | >75,000 persons trained by BRAC including community volunteers, CHWs, nurse midwives, TBAs, NGO program managers, supervisors, govt. and NGO health assistants, and female health workers, national master trainers, regional trainers, govt. health and family planning managers and staff |
| Saha KK, et al 2011. Alive & Thrive Baseline Survey Report: Bangladesh. Washington, D.C.: FHI360, IFPRI | The baseline survey includes a census, household survey, community survey, and frontline health workers survey; conducted in 20 subdistricts across five of the country’s six regions. | Mothers needed counseling and support from health workers for perceptions that children disliked solid foods, poor appetite, and perception of breast milk insufficiency. Knowledge was high while practices were poor. | The need for changing maternal knowledge into practices on IYCF through motivation, social support, self-efficacy using multiple influential persons and sources of information. |
| Saha KK, et al 2014, Bangladesh Impact Evaluation Final Report: Bangladesh. Washington, D.C.: IFPRI, 2014. See Annex A2.1 Program Implementation Pathway of the Community Component A2.2 Program Implementation Pathway of the Communication Component. | Cluster randomized evaluation of impacts on IYCF and anthropometric outcomes, conducted in 20 subdistricts across five of the country’s six regions. | Identified the multiple channels and activities through which mothers were reached | Identified categories of influentials reached and the multiple channels and activities through which influentials were reached. |
| Rasheed S, et al, 2011 Why Does Nutrition Deteriorate Rapidly among Children under 2 Years of Age? Using Qualitative Methods to Understand Community Perspectives on Complementary Feeding Practices in Bangladesh. | Data from two rural and one urban location in through semi structured interviews, food attributes exercises, 24-hour dietary recalls, opportunistic observations, and trials of improved practices (TIPs). Key informant interviews and focus group discussions among family members and community opinion leaders. Food and Nutrition Bulletin. 2011;32(3):192-200. | Need for counseling mothers and those who influence them was identified. | Complementary feeding advice from family members, peers, and health workers, the importance given to feeding young children by family decision makers, and time spent by caregivers in feeding influenced the timing, frequency, types of food given, and ways in which complementary feeding occurred. |
| Frongillo EA. 2020. Designing and implementing at-scale programs to improve complementary feeding. | Review paper on three large scale initiatives to improve CF practices. Nutr Rev. 2020;78(Suppl 2):62-70 | By mid-2014, the program had reached about 1.7 million mothers and children under the age of 2 years. | Training of more than 75 000 frontline workers and health providers throughout the country. Monthly home visits involved engaging other family members. |
| **Ethiopia** | | | |
| Kim et al, 2016. Exposure to Large-Scale Social and Behavior Change Communication Interventions Is Associated with Improvements in Infant and Young Child Feeding Practices in Ethiopia. | External cluster randomized evaluation of IYCF practices and coverage using cross-sectional surveys at baseline and endline 4 years later. PLoS One. 2016;11(10):e0164800 | About 92% of women with children less than two years of age had been exposed to at least one intervention channel in the in the six months preceding the endline survey, and over half of the mothers had been exposed to 2–3 channels. During the intervention period, the A&T model reached large scale with an estimated 1.5 million mothers of children under two years exposed to IPC across 295 intervention woredas (districts). An estimated 960,000 women heard the A&T radio spots, for an estimated total of 2 million mothers of children under two years reached by either IPC or mass media. | Training was provided to the Federal Ministry of Health’s flagship Health Extension Program, to female health extension workers (HEWs) and cadres of community health volunteers known as the Women’s Development Armies (WDAs) or Health Development Armies (HDAs). Community mobilization and mass media interventions were directed at opinion leaders, fathers and other caregivers through training and materials for local organizations such as faith-based organizations and women’s associations. |
| Frongillo EA. 2020. Designing and implementing at-scale programs to improve complementary feeding. | Review paper on three large scale initiatives to improve CF practices. Nutr Rev. 2020;78(Suppl 2):62-70 | The program was implemented by multiple partners in the 4 most populous regions of the country. The messages and counseling were delivered primarily through the large network of government-salaried female health extension workers and community health volunteers referred to as the Women’s Development Armies or Health Development Armies. The interpersonal counseling took place at health posts or during routine home visits. | Community mobilization activities such as village gatherings and food demonstrations were carried out, along with a mass media campaign primarily relayed via radio. These activities reached opinion leaders, fathers, and other caregivers as well as mothers. |
| Kim SS, et al, 2015. Assessing implementation fidelity of a community-based infant and young child feeding intervention in Ethiopia identifies delivery challenges that limit reach to communities | Mixed-method process evaluation study. BMC Public Health. 2015;15(1):316. | Variability between regions could limit the potential for impact on mothers. Strengthening the linkages between HEWs and volunteers can help to reach the target households and deliver IYCF results at scale. | Strong fidelity in training and delivery of program tools and messages at higher FLW levels, but low reach to community volunteers who were counseling mothers (54% of volunteers in Tigray and 39% in SNNPR). Quality of program message delivery was low among volunteers, and recall of key messages among mothers was also low. Although FLW supervision was high, content and frequency were irregular. |
| A&T, 2010, Formative research report, Tigray | Semi-structured interviews, focus groups discussions and observations in Tigray Region’s central, eastern, and southern zones with mothers, family members, health workers and community members. | The need for counseling mothers and training of health personnel to conduct skilled counselling on IYCF was identified. | Mothers get direct or indirect advice and support from various sources such as health extension workers, volunteers, and their families. |
| Ali D, et al. 2011. Alive & Thrive Baseline Survey Report Executive Summary: Ethiopia. Washington, D.C.: Alive & Thrive, FHI 360, IFPRI | The baseline survey included a household survey, a frontline health worker survey, and community survey. A total of 3,000 households were selected from 56 high population districts (19 in Tigray and 37 in SNNPR regions). | Mothers needed counseling on to their child being sick and their child refusing to eat. Two thirds of the mothers reported seeking help for these problems. Only 15 percent had heard messages on IYCF. | Frontline health workers and older female members are the primary source of support when women were facing difficulty in feeding. |
| **Vietnam** | | | |
| Rawat R, et al. 2017. Social Franchising and a Nationwide Mass Media Campaign Increased the Prevalence of Adequate Complementary Feeding in Vietnam | Cluster-Randomized Program Evaluation. J Nutr. 2017;147(4):670-9 | Mothers with children aged 6–23.9 mo who reported ever being exposed to IPC at a program health facility was 41.9%. TV coverage was 36% in program and 31% in control areas. | Influentials reached include health workers trained to provide IPC, village volunteers to motivate mothers to seek IPC, and mass media estimated to have reached >11 million women aged 15–35 y (media monitoring reports). |
| Nguyen TT, et al. 2021 Community support model on BF and CF feeding practices in remote areas in Vietnam. | Review of reports, expenditure and coverage data, monitoring data, and budgets in 9 provinces, a cross-sectional survey in 3 provinces. Int J Equity Health. 2021;20(1):121. | 801 support groups in 267 villages implemented; facilitators provided ~ 166,000 meeting/support contacts with ~ 33,000 pregnant women and mothers with children. | Support group facilitators and trainers engaged in the program, community leaders and government staff oriented. |
| Nguyen P, et al. 2011. Alive & Thrive Baseline Survey Report, FHI 360, IFPRI | Conducted in 40 communes across four provinces, Thai Nguyen, Thanh Hoa, Quang Ngai, and Vinh Long; includes household surveys, community questionnaires, frontline health worker surveys, and health facility assessments. Viet Nam. Alive & Thrive, 2011. | Mothers needed counseling on initiating complementary feeding and dealing with poor appetite and feeding during illness. They have good access to the government health system; 70 percent of them reported watching television daily. | Mothers/mothers-in-law or family members and healthcare providers played an important role in providing support and information for mothers. |
| Nguyen at al, 2019. Intervention Design Elements Are Associated with Frontline Health Workers’ Performance to Deliver Infant and Young Child Nutrition Services in Bangladesh and Vietnam | Survey data from 2010 and 2014 used for multiple regression to test differences in FLW exposure and performance outcomes; path analyses to track exposure links to performance outcomes in FLW and end-users. Curr Dev Nutr 2019;3:nzz070. | NA | Health workers, village volunteers, and managers in program centers and catchment areas were trained to increase IYCF counseling or IPC. Additionally, mass media reached a number of influentials through multiple channels; evaluation of health workers exposed to mass media showed improved performance scores. |
| Nguyen, Phuong H. et al. 2014. Program Impact Pathway Analysis of a Social Franchise Model Shows Potential to Improve Infant and Young Child Feeding Practices in Vietnam | Mixed methods including qualitative interviews with franchise management board members (*n* = 12), surveys with health providers (*n* = 120), counseling observations (*n* = 160), and household surveys (*n* = 2045). J Nutrition, Volume 144, Issue 10, 1627 - 1636 | Need for improved routine monitoring to track IYCF counseling in mothers. Survey showed franchise utilization increased from 10% in 2012 to 45% in 2013 | Training was associated with increased capacity of providers, resulting in higher-quality IYCF counseling (greater technical knowledge and communication skills during counseling). |
| A&T Brief on Formative Research on IYCF in Vietnam. 2012. Phase 1 | Qualitative and quantitative studies in 16 communes across 9 provinces on practices and determinants. A&T Form.-VN-Phase-1-summary_17_Jun_2012.pdf | Counseling needed for mothers on too early CF, low nutrient density, poor hygiene practices. High TV coverage. | Health workers and grandmothers identified as key influentials. |

Acronyms: A&T=Alive and Thrive, BRAC= national development NGO of Bangladesh, CF= complementary feeding, CHW=community health worker, FLW=front line worker, IFPRI=International Food Policy Research Institute, IPC=interpersonal communication, MNCH=maternal, newborn and child health, NA=not available, NGO=non-governmental organization, TBA=traditional birth attendant
